# Becoming as Open‐Minded and Organized as My Classmates? Peer Effects on Self‐Reported Personality Trait Development in the Classroom

**DOI:** 10.1111/jopy.13009

**Published:** 2025-01-10

**Authors:** Mieke Johannsen, Naemi D. Brandt, Oliver Lüdtke, Jenny Wagner

**Affiliations:** ^1^ Department of Psychology University of Hamburg Hamburg Germany; ^2^ Department of Psychology Kiel University Kiel Germany; ^3^ Leibniz Institute for Science and Mathematics Education at Kiel University Kiel Germany; ^4^ Centre for International Student Assessment Munich Germany

**Keywords:** adolescence, classroom peers, composition effect, multilevel structural equation modeling, personality development

## Abstract

**Introduction:**

How does a student's personality development relate to the personality of their classmates? The school class builds a pertinent comparison group during adolescence that has been identified as a critical factor in students' development of abilities and self‐perceptions. This study empirically tests the impact of classroom personality composition on changes in adolescents' Big Five personality traits. We hypothesized positive associations between class‐level openness and conscientiousness and the individual development of these traits given their role in academic performance.

**Method:**

To test these hypotheses and explore additional composition effects, we employed three approaches of multilevel structural equation modeling on two large longitudinal samples of German adolescents (*N*_1_ = 5470; *N*_2_ = 788).

**Results:**

Our analyses yielded two principal findings: First, individual personality levels remained highly stable across different time periods. Second, contrary to our hypotheses, baseline class‐level openness and conscientiousness were not positively linked to individual personality development. Instead, there were some indications that higher class‐level openness was negatively linked to individual openness to experiences at the second measurement point.

**Conclusions:**

We discuss the absence of systematic composition effects at the classroom level and consider methodological challenges in investigating these effects.

To what degree does the way students see themselves relate to their classmates? How people see themselves is reflected in their personality, defined as relatively stable individual differences in how people think, act, and feel (Roberts 2009). Often, we use people as informants on their personalities by asking them to self‐report their typical behaviors, thoughts, and feelings. However, according to social comparison theories (e.g., Festinger 1954; Wood et al. 2012), such self‐perceptions involve comparisons to others (besides potential other sources; McCrae 2018). Accordingly, personality self‐reports have been found to vary with the given reference group instructions (Credé, Bashshur, and Niehorster 2010; Lenhausen, Bleidorn, and Hopwood 2023; Lenhausen, Hopwood, and Bleidorn 2023). Specifically, people of the same age appear to form the most relevant reference group for personality self‐reports in adulthood (Lenhausen, Hopwood, and Bleidorn 2023).

In adolescence, schools and classrooms constitute institutionalized peer groups that are not self‐selected and entail frequent social interactions with same‐aged peers (Eccles and Roeser 2011). Therefore, educational contexts allow for a meaningful investigation of peer effects (Marsh et al. 2020). Research on composition effects has argued that peers are not only relevant comparison targets but also mold an individual's academic behaviors and specific self‐perceptions (e.g., Fang et al. 2018; Van Ewijk and Sleegers 2010). That is, the average level of certain peer characteristics within a classroom should be associated with an individual's development in the same characteristic. To illustrate, a student might adapt their level of diligence and organization across time when being in a classroom with more diligent and organized classmates. While the same student might show and report less diligence and organization within a less organized class. However, so far we know little about the degree to which an adolescent's Big Five personality development is associated with their classmates' average personality manifestations—despite the relevance of several personality traits within the educational context (e.g., Israel, Lüdtke, and Wagner 2019; Mammadov 2022).

Building on the relevance of peers for personality self‐reports of adults (Lenhausen, Hopwood, and Bleidorn 2023) and the established knowledge regarding the relevance of class‐average peer characteristics in the school context (e.g., Fang et al. 2018; Van Ewijk and Sleegers 2010), the current study sets out to integrate these two lines of research to provide a first empirical test of classroom peer effects on adolescents' Big Five personality self‐reports. By applying multilevel structural equation modeling to two samples of *N* = 5470 and *N* = 788 German secondary school students, we test the assumption that the development in self‐reported personality traits is associated with the initial average level of personality traits of their classmates.

## 1 Reference Group Effects on Personality Self‐Reports

As a framework for relatively stable individual differences, the Big Five (Costa and McCrae 1992) offer a comprehensive conceptualization to describe people's personalities. The assessment of the five personality traits of openness, conscientiousness, extraversion, agreeableness, and neuroticism (or emotional stability) commonly involves self‐report scales (e.g., Ostendorf and Angleitner 2004; Soto and John 2017). Self‐report questionnaires using Likert scales—the typical scale format of (personality) questionnaires—do not objectively capture one's absolute level of a given trait but rather require the respondent to form a self‐evaluation of the assessed characteristic (Heine et al. 2002). Social comparison theories have argued that these self‐evaluations involve comparisons to reference groups (e.g., Festinger 1954). For example, there is no objective consensus on a threshold for being considered industrious. Rather the reported level of industriousness depends on comparisons with a given or chosen reference group. Personality research has only very recently considered such reference group effects more closely focusing on the kind of reference groups used when people report their personality (Heine et al. 2002; Lenhausen, Bleidorn, and Hopwood 2023; Lenhausen, Hopwood, and Bleidorn 2023).

The first studies on reference group effects on personality self‐reports stem from cross‐cultural research (e.g., Heine et al. 2002). These studies highlight the relevance of cultural contexts, norms, and expectations for forming self‐evaluations assessed with personality self‐reports. However, more recent studies on personality assessments also point to the relevance of more specific comparison groups (Credé, Bashshur, and Niehorster 2010; Lenhausen, Bleidorn, and Hopwood 2023; Lenhausen, Hopwood, and Bleidorn 2023). To illustrate this relevance, Lenhausen, Bleidorn, and Hopwood (2023) and Lenhausen, Hopwood, and Bleidorn (2023) systematically varied their instructions to examine potential reference group effects in adult samples. More specifically, they asked participants to report their personality relative to different reference groups: on a between‐person level, participants were asked to compare themselves to people in general, close others, people their age, and people the same gender, whereas, on a within‐person level, they were asked to compare themselves to their ideal self and their past self. Furthermore, participants also reported what they believed they most strongly used as a reference group if none was specified. Findings suggest that while most people believed to use *people in general* as a reference, the most influential reference group was people the *same age* as the participants. Thus, same‐aged peers form a relevant reference group for personality self‐reports in adulthood. However, we know little about *how* people perceive and describe their levels of Big Five personality relative to same‐aged peers when it comes to adolescence and the specific context of the school class.

## 2 Classrooms as Reference Groups for Personality Development

Investigating the relevance of peers for personality self‐reports is particularly interesting in adolescence because adolescents face (among others) two developmental tasks that seem to be important in the context of peer effects on personality (Eccles et al. 1989; Erikson 1968; Klimstra 2013). First, adolescents need to develop a stable picture of themselves as a person, whereby personality traits are still comparably unstable during the developmental phase of adolescence (Soto and Tackett 2015). Second, adolescents expand their networks beyond the family and peer relationships become increasingly relevant (Cillessen and Borch 2006; Smetana et al. 2006; Wagner et al. 2014). Moreover, schools and classrooms are the most prevalent developmental contexts in adolescence (Eccles and Roeser 2011). Classrooms offer frequent peer interactions and constitute clearly defined institutional peer groups (Marsh et al. 2020). This is especially true within the German school system, where classes often are not subject‐specific but students in one class regularly go through their entire curriculum together over several years (Becker, Neumann, and Dumont 2017). Along these lines, classrooms are specifically suitable to address the role of peers in an individual's self‐perceptions of personality.

Various disciplines have addressed the relevance of peer composition within educational contexts (cf., Van Ewijk and Sleegers 2010). As a general proposition, composition effects in education assume that the average characteristic of an entire school class relates to an individual student's learning outcomes and development of this characteristic (Hattie 2002). Empirically, educational researchers have shown that an individual's performance varies with the composition of schools and classes regarding different peer characteristics, such as socioeconomic status (e.g., Van Ewijk and Sleegers 2010) and performance (Becker et al. 2022). That is, attending a socioeconomically well‐positioned school or class is associated with better performance, as is attending a school or class with a higher average performance. Moreover, research indicates that classroom composition is not only linked to students' performance but also their socioemotional characteristics like well‐being (Belfi et al. 2012) and ability self‐perceptions (Fang et al. 2018). Interestingly, while class‐average achievement is positively related to individual achievement and its development (Becker et al. 2022), it shows negative associations with individual ability self‐perceptions (Fang et al. 2018). These findings might reflect the different processes involved in composition effects (Dreeben and Barr 1988; Thrupp, Lauder, and Robinson 2002). To specify, beyond social comparison processes which suggest a negative association of the class level with individual levels of a specific characteristic, it is likewise possible that students internalize the norms of their peer group and adapt their learning and behavior (in the sense of a socialization process) resulting in a positive link between the class and individual level.

While there is support for the relevance of social comparison processes for personality self‐perceptions (Credé, Bashshur, and Niehorster 2010; Lenhausen, Bleidorn, and Hopwood 2023; Lenhausen, Hopwood, and Bleidorn 2023), there is also a first study supporting peer socialization effects for Big Five trait development (Shan and Zölitz 2023). This study from the field of economics was based on a higher education sample in small learning groups (four students) and indicates that group‐average peer personality positively relates to an individual's personality development (Shan and Zölitz 2023). Specifically, being randomly assigned to a learning group with more open‐minded and conscientious peers increased individual levels of these traits across one semester within the selective academic environment of a Swiss private university. However, due to the highly selective private university setting and the small peer group, it is an open question whether these positive personality peer effects generalize, or whether negative comparison effects similar to those related to ability self‐perceptions occur in a more diverse adolescent population and larger classrooms.

Integrating these different lines of research, we expect that the class level is associated with an individual's development of the respective Big Five trait. However, given their differential role in academic outcomes (e.g., Israel et al. 2023; Mammadov 2022), we expect different associations for specific traits. We argue for a particularly straightforward relevance of classroom peers regarding the two traits of openness and conscientiousness. Research has demonstrated that both traits relate to everyday school behavior and academic performance (e.g., Israel et al. 2023; Mammadov 2022). Students high in openness and, to a somewhat stronger extent, those high in conscientiousness show better academic performance in the classroom (Mammadov 2022; Poropat 2009). Taking up the results from Shan and Zölitz (2023) indicating socialization processes, we argue that students adapt to the typical class‐average of open and conscientious behavior within their class. Therefore, self‐perceptions of openness and conscientiousness within classrooms should be positively linked to the average level of these traits across all classroom peers. That is, we assume that higher initial class‐level averages of openness and conscientiousness are associated with an increase in the individual student level of self‐reported openness and conscientiousness across time spent in the classroom. To illustrate, an intellectually less curious student surrounded by many intellectually curious students (higher class level of openness) might adapt and show more intellectually curious behavior. However, the same student surrounded by only a few intellectually curious students (lower class level of openness) might experience a decrease in their self‐reported level of openness.

Although extraversion, agreeableness, and neuroticism are less consistently associated with performance indicators in the school context (Meyer et al. 2023), they have been related to positive social interactions in adolescence inside and outside of the classroom (e.g., Israel et al. 2023; Wieczorek et al. 2022). High levels of extraversion relate to higher likeability and popularity (De Vries et al. 2020; Hubers et al. 2016; Van Der Linden et al. 2010) and friendship development in the school context (Israel et al. 2023). Higher levels of agreeableness are associated with better peer (and teacher) cooperation (Miller, Lynam, and Leukefeld 2003) and larger social networks (De Vries et al. 2020). Higher levels of neuroticism in adolescence are associated with insecurity (Deventer et al. 2019), lower levels of emotional closeness (Wagner et al. 2014), and lower popularity among classmates (Van Der Linden et al. 2010). Along these lines, extraversion, agreeableness, and neuroticism appear to be more of a social as compared to a performance function and, thus, might be less prone to be affected by performance‐related peer interactions within classrooms. This might explain the absence of peer effects on these traits within university learning groups (Shan and Zölitz 2023) that may not serve as much of a social function as the classroom context. Due to the particular importance of social relationships with peers during adolescence and the relevance of social interactions within classrooms, we assume the classroom levels of extraversion, agreeableness, and neuroticism relate to self‐reported individual levels of these traits. However, given the lack of previous studies in educational contexts indicating whether social comparison or socialization processes are more relevant for the development of these traits, we investigate the direction of these peer group effects exploratory.

## 3 The Present Study

Given the relevance of peers for personality self‐reports (Lenhausen, Hopwood, et al. 2023), the established knowledge regarding classroom peers affecting an individual's learning outcomes (Fang et al. 2018; Van Ewijk and Sleegers 2010), and the relevance of personality within classroom contexts (Israel, Brandt et al. 2023), the current study sets out to provide a first empirical test of peer composition effects on adolescents' self‐reported Big Five personality in the classroom context. Specifically, we assume that the initial class‐level average of openness (H1a) and conscientiousness (H1b) relate positively to an individual's development of the respective traits controlling for initial individual levels. We test composition effects on the remaining three traits exploratorily. To test our hypotheses, we apply multilevel SEM to two samples of *N* = 5470 and *N* = 788 of German secondary students.

## 4 Transparency and Openness

We preregistered our hypotheses and analytic strategy at the OSF (https://osf.io/evga9/) before data analysis and provided a comprehensive supplement. All analysis codes, Supporting Information, and data of Study 2 are also available on the OSF project page. We prepared all data in R (4.3.3; R Core Team 2024) and subsequently estimated the MLM models testing our research questions in M*plus* (8.5.; Muthén and Muthén 2021) by means of the M*plus*Automation package (1.1.0; Hallquist and Wiley 2018). The analyses were performed in line with our preregistered analysis plan except for some deviations documented in Table S1 at the OSF. We follow JARS (Appelbaum et al. 2018) in reporting our results. We addressed our research questions based on two independent already existing datasets. The dataset of Study 1 stems from the German National Educational Panel Study (NEPS) and data is open source and available on the NEPS project page (https://www.neps‐data.de/Data‐Center/Data‐Access). The dataset of Study 2 is part of the SEED project and is uploaded to the OSF project page.

We determined a lower boundary^1^ for power by conducting a series of simulations with the R‐packet *simr* (Green and MacLeod 2016), following the tutorial by Arend and Schäfer (2019). Simulation results suggest that the size of the NEPS sample (*N* = 5470 nested in *k* = 606 classes)^2^ provides sufficient power to detect small (*b* = 0.10) student‐level effects and small to medium (*b* = 0.20) class‐level effects. Regarding the SEED sample (*N* = 788 nested in *k* = 54 classes), simulation results suggest that the given sample size provides sufficient power to detect small to medium (*b* = 0.20) student‐level effects and medium (*b* = 0.50) class‐level effects.

## 5 Study 1: Method

Within Study 1, we used publicly available data from the German National Educational Panel Study (NEPS), starting cohort 3. The NEPS is carried out by the Leibniz Institute for Educational Trajectories (LIfBi, Germany) in cooperation with a nationwide network. Further information on the NEPS data is available at https://www.neps‐data.de/Mainpage. It provides representative longitudinal data from German samples across six different age cohorts (for details, see Blossfeld et al. 2011). Given our interest in adolescent personality development, we based our analyses on data from starting cohort 3 (NEPS Network 2023) as it provides personality data collected in the 7th (T1) and 9th (T2) grades. More specifically, for T1 we took wave 3 data, and for T2 we combined waves 5 and 6, which both have been assessed in 9th grade.

### 5.1 Sample

Out of the 7805 NEPS participants available within starting cohort 3 who attended regular schools, we, first, excluded all participants who did not report any personality data at T1 (*n* = 1135), second, all participants for whom there was no class identification available at any time point (*n* = 660), and, finally, all participants that changed classes in between measurements (*n* = 540).^3^ The exclusion of a total of *n* = 2335 participants resulted in a total sample of *N* = 5470 seventh graders nested within 606 classes across 272 schools for Study 1. There were *M* = 9.02 (*SD* = 5.90; range^4^: 1–31) participants in each class on average. Participants were equally distributed across vocational‐track schools (51.4%), which prepare for vocational training, and academic‐track schools (48.6%), which prepare students for university education. On average, students were 12.86 (*SD* = 0.52) years old at T1, with an equal distribution of girls and boys (48.3% female). Looking at the SES of parents, the average HISEI was 56.79 (*SD* = 19.84) ranging between 11.74 and 88.96. An immigrant background was reported by 32.0% of the participants.

A selectivity analysis indicated that compared to the *n* = 2335 excluded students, the *N* = 5470 participants included in our analyses showed higher fluid reasoning scores (*t*(3636.9) = 7.10, *p* < 0.001, *d* = 0.21), were less likely to have a migration background (*t*(3923.6) = −3.65, *p* < 0.001, *d* = 0.11), and came from families with higher SES (*t*(2784.1) = 3.89, *p* < 0.001, *d* = 0.12). There were no significant differences in the personality scale scores at the first measurement between included and excluded participants (all *p*s > 0.175).

### 5.2 Measures

#### 5.2.1 Big Five Personality Traits

Big Five personality traits were measured using the BFI‐10 (Rammstedt and John 2007). The BFI‐10 measures the Big Five dimensions with two items each, one of which is reverse‐keyed to control for acquiescent responding. Following the suggestion of Rammstedt and John (2007), NEPS included a third agreeableness item to improve the reliability and bandwidth of the agreeableness scale of the BFI‐10. We used these three agreeableness items in our analyses. All personality items were rated on a fully labeled 5‐point scale ranging from 1 (*does not apply at all*) to 5 (*fully applies*). We included personality measures from T1 and T2 in our analyses. In line with other studies (Brandt et al. 2020; Brandt and Lechner 2022) and due to the low number of items for such broad constructs, the split‐half reliabilities of the manifest scales were relatively low (*r*_opennessT1/T2_ = 0.36/0.48, *r*_*c*onscientiousness T1/T2_ = 0.53/0.50, *r*_extraversion T1/T2_ = 0.40/0.62, *r*_agreeableness T1/T2_ = 0.40/0.36, *r*_neuroticism T1/T2_ = 0.33/0.44). However, the test–retest reliability of the BFI‐10 is sufficient as reported in previous research in Germany (Rammstedt et al. 2020). Moreover, it shows high convergent validity with longer Big Five scales and the overall criterion validity is comparable to longer inventories (Rammstedt and John 2007; Thalmayer, Saucier, and Eigenhuis 2011). To account for the unreliability of the observed items, we used latent variable models for all Big Five traits.

#### 5.2.2 Control Variables

Control variables are all considered from T1. *Gender* (0 = male, 1 = female), *fluid reasoning*, and parental *socioeconomic status* served as manifest control variables at the individual level. *School track* (0 = nonacademic; 1 = academic) served as a control variable at the class level. Within the NEPS, fluid reasoning was measured by the 12‐item matrices test (NEPS‐MAT). NEPS‐MAT is similar to Raven's Standard Progressive Matrices and was developed and validated specifically for the NEPS (for further information, see Brunner, Lang, and Lüdtke 2014) and socioeconomic status was indicated by parents' highest International Socio‐Economic Index of Occupational Status (HISEI; Ganzeboom, De Graaf, and Treiman 1992).

### 5.3 Statistical Analyses

Given our interest in effects of average classroom characteristics and to account for the nested structure in our data, that is students (L1) nested in classrooms (L2), we estimated a set of multilevel structural equation models (multilevel SEM). Our multilevel SEMs consisted of three main components: Student personality at the second measurement point (T2) was predicted by personality at T1 (L1), by the average classroom personality at T1 (L2), and by a subsequently added set of covariates (L1 and L2). Given our research question, the main interest is on the average classroom personality effects, which in this modeling approach is called a composition effect (Lüdtke et al. 2008). A composition effect is regarded to be present if the average classroom personality variable on level 2, which is based on the aggregated individual‐level personality scores within one classroom, is related to individual personality at T2 over and above the effect of personality at T1. In order to assess the robustness of our estimates of composition effects to different modeling strategies, we decided to apply three modeling strategies (Lüdtke et al. 2008; Marsh et al. 2009): (1) doubly latent contextual models, (2) manifest‐latent contextual models, and (3) the Bayesian modeling approach to estimate double‐latent contextual models (Depaoli 2021; Zitzmann et al. 2016).^5^

First, we estimated doubly latent contextual models (Lüdtke et al. 2008; Marsh et al. 2009) using the robust maximum likelihood estimator (MLR; Yuan and Bentler 2000). These models allow to control for both measurement error at the individual level and sampling error in the classroom level variable. To control for measurement error, we specified the Big Five traits as latent constructs. More specifically, given the small number of items (two or three items per trait) for personality in the NEPS data, we fixed the loadings within each factor to 1.^6^ Furthermore, we allowed for correlated item residuals across time. To control for sampling error, the unobserved group mean is regarded as a latent variable that is measured with a certain amount of precision by the group mean of the observed data (e.g., latent aggregation). Moreover, we accounted for the sampling of classrooms within schools (L3) by correcting the standard‐error estimation for the nested data structure (analysis option type = complex two‐level in Mplus). To handle missing values, we used the full information maximum likelihood estimation (Enders 2010).

Second, we exploratively estimated manifest‐latent contextual models (Lüdtke et al. 2008; Marsh et al. 2009). To avoid modeling issues referring to the latent measurement of the Big Five traits (i.e., negative error variances), we decided to estimate manifest‐latent contextual models. That is, instead of specifying latent measurement models, we used the total scale scores as manifest indicators of each trait, while keeping all other model specifications identical to our first modeling approach. Thus, these models do not control for potential measurement error, however, they, nevertheless, include a latent aggregation of the trait at the class level and thus control for potential sampling error.

Third, we specified double‐latent contextual models, but used a Bayesian modeling approach (Zitzmann et al. 2016). Double‐latent contextual models have been linked to estimation problems such a inadmissible results (i.e., negative variances) especially in contexts with little information on the group‐level (e.g., low ICCs; Zitzmann et al. 2016). According to simulation studies, the Bayesian estimation approach leads to fewer estimation problems and additionally increases the accuracy of the estimates of group‐level effects in these cases (Depaoli 2021; Zitzmann et al. 2016). We largely followed the approach of Zitzmann et al. (2016) and specified our priors accordingly. More specifically, the Bayesian approach allowed us to avoid negative residual variances without having to restrict them to 0 by specifying a weakly informative prior for the between‐group level. Detailed model syntax are uploaded at the OSF project page (https://osf.io/evga9/).

Following conventional guidelines (Hu and Bentler 1999; Schermelleh‐Engel, Moosbrugger, and Müller 2003), we based our model evaluations for the first and second approach on several criteria: the confirmatory fit index (CFI < 0.95 or at least < 0.90), root mean square error of approximation (RMSEA < 0.05 or at least < 0.08), and standardized root mean square residual (SRMR < 0.05 or at least < 0.10). All variable preprocessing was done with R (R Core Team 2024) using R‐Studio and all multilevel SEMs were estimated using Mplus (Muthén and Muthén 2021) by means of the *MplusAutomation* package (Hallquist and Wiley 2018). We standardized all continuous variables across the sample to facilitate the interpretation of the regression coefficients from multilevel SEMs. Please also see our model outputs at the OSF (https://osf.io/evga9/) for complete model syntaxes for all modeling approaches. To account for the number of models and estimates in our analytic procedure, we will only interpret findings being significant on the *p* < 0.01 level. We also report exact *p*‐values and 99% confidence intervals (credibility intervals for Bayesian estimates) for all parameter estimates to provide the reader with complete information.

## 6 Study 1: Results and Discussion

Intraclass correlation coefficients (0.01–0.03) indicated a low amount of variance in the personality scales scores at T1 being attributable to the class level (see Table 1). To illustrate, an ICC of 0.03 for the trait of openness suggests that only 3% of the total variance in openness scores can be attributed to the classroom level, while 97% of the variance is due to individual differences between students within the same classes. Thus, across all Big Five personality traits most variance and interindividual differences can be found between students within one classroom but less so between classrooms. Table 2 shows manifest correlations between the Big Five personality traits at both measurement points and all covariates.

**TABLE 1:** Study 1—intraclass correlation coefficients (ICCs) and cluster variances for personality scales.

	ICC	var_within_	var_between_
Openness T1	0.03	0.89	0.03
Conscientiousness T1	0.02	0.72	0.02
Extraversion T1	0.02	0.60	0.01
Agreeableness T1	0.03	0.41	0.01
Neuroticism T1	0.01	0.68	0.01

Abbreviations: T1, first measurment in 7th grade; var_between_, variance between classes; var_within_, variance within classes.

**TABLE 2:** Study 1—means, *SD*s, number of observations, and correlations of big five traits, socio‐demographic characteristics, and covariates.

Variable	*M*	*SD*	*N*	1	2	3	4	5	6	7	8	9	10	11	12	13	14	15	
1. Openness T1	3.44	0.96	5382																
2. Openness T2	3.36	0.95	4332	**0.51**															
3. Conscientiousness T1	3.22	0.86	5381	**0.10**	**0.05**														
4. Conscientiousness T2	3.05	0.83	4324	**0.05**	**0.08**	**0.51**													
5. Extraversion T1	3.40	0.79	5302	**0.08**	**0.06**	0.00	0.01												
6. Extraversion T2	3.30	0.85	4324	0.02	**0.05**	0.03	**0.07**	**0.49**											
7. Agreeableness T1	3.45	0.65	5307	**0.19**	**0.10**	**0.30**	**0.17**	−0.00	−0.00										
8. Agreeableness T2	3.44	0.64	4305	**0.09**	**0.13**	**0.17**	**0.18**	−0.02	−0.02	**0.38**									
9. Neuroticism T1	2.83	0.83	5353	0.02	−0.01	**−0.04**	−0.03	**−0.23**	**−0.19**	−0.02	**0.05**								
10. Neuroticism T2	2.84	0.85	4326	**0.07**	0.03	−0.00	−0.02	**−0.13**	**−0.29**	**0.04**	0.01	**0.37**							
11. Female	0.48	0.50	5466	**0.22**	**0.21**	**0.17**	**0.14**	0.03	−0.02	**0.18**	**0.13**	**0.17**	**0.28**						
12. HISEI	56.79	19.84	3689	**0.08**	**0.06**	0.04	−0.00	**0.08**	0.04	0.03	−0.00	**−0.05**	−0.03	0.01					
13. Fluid Reasoning T0	7.08	2.58	3340	**0.10**	**0.07**	−0.04	**−0.12**	0.00	−0.04	−0.00	0.00	**−0.05**	**−0.07**	−0.04	**0.21**				
14. Fluid Reasoning T2	9.19	2.25	3849	**0.10**	**0.09**	0.01	**−0.06**	−0.02	−0.03	0.03	0.03	**−0.05**	−0.03	0.01	**0.23**	**0.50**			
15. Immigrant Background	0.32	0.47	3333	0.02	**0.06**	0.01	0.02	0.02	0.02	0.02	−0.00	0.01	0.01	0.03	**−0.16**	**−0.12**	**−0.12**		
16. School Track	0.49	0.50	5450	**0.09**	**0.06**	0.03	**−0.07**	**0.08**	**0.06**	0.02	−0.02	**−0.06**	−0.02	**0.04**	**0.39**	**0.37**	**0.35**	**−0.07**	

*Note:* Significant correlations (*p* < 0.01) are in bold. Sample sizes vary across correlations from 2453 to 5466 due to missing values. HISEI = parents' highest International Socio‐Economic Index of Occupational Status (Ganzeboom, De Graaf, and Treiman 1992). Immigrant background is dummy coded with 1 = immigrant background. School track is dummy coded with 1 = academic track.

In the following, we report and compare the results from the three modeling approaches introduced above: doubly latent contextual models, manifest latent models, and Bayesian estimation of doubly latent contextual models. Across modeling approaches, our models with and without covariates fit the data well (see Table S2 for detailed information on model fit). We report all findings with and without covariates (gender, fluid reasoning ability, and socioeconomic status at the individual, school track at the class level) and thus, illustrate if our results are robust to the inclusion of covariates.

Regarding the rank‐order stability of the Big Five traits at the individual level, results generally showed high stability coefficients. Given the latent variable models control for measurement error, the size of the estimates of the latent modeling approaches (*b*s: 0.60–1.04, *p*s < 0.001) was expectedly larger compared to the manifest‐latent approach (*b*s: 0.38–0.51, *p*s < 0.001). This result pattern remained widely identical once we added the covariates (see Tables S3 and S4). Taken together, personality showed to be relatively stable from 7th to 9th grade.

Regarding the composition effects, regression coefficients obtained from the different modeling approaches are shown in Table 3. Across modeling approaches there were almost no significant compositions effects. Specifically, there were no composition effects in any of the manifest‐latent models and the doubly latent models using a Bayesian approach, but there was one composition effect in the doubly latent models using the maximum‐likelihood approach. That is, the composition effect of openness reached statistical significance (*b* = −0.33, *p =* 0.009) and this effect was robust for the inclusion of covariates (*b* = −0.42, *p =* 0.008). Contrary to our hypothesis, the composition effect of openness was negative indicating that a higher class‐average of openness at the first measurement point was associated with lower individual levels of openness at the second measurement point. Thus, in classrooms with higher average levels of openness in 7th grade, students reported lower levels of openness 2 years later in 9th grade controlling for the individual stability.

**TABLE 3:** Study 1—composition effect estimates obtained across modeling approaches.

	Doubly Latent			Manifest Latent			Bayesian		
	*b*	*p*	99% CI	*b*	*p*	99% CI	*b*	*p*	99% CI
Openness									
Uncontrolled	**−0.33**	0.009	[−0.65, −0.00]	0.07	0.537	[−0.21, 0.35]	−0.31	0.020	[−0.68, 0.08]
Controlled	**−0.42**	0.008	[−0.83, −0.01]	−0.05	0.748	[−0.42, 0.33]	−0.39	0.021	[−0.90, 0.12]
Conscientiousness									
Uncontrolled	−0.23	0.283	[−0.78, 0.32]	0.03	0.905	[−0.54, 0.59]	−0.12	0.267	[−0.62, 0.38]
Controlled	−0.03	0.900	[−0.61, 0.55]	0.22	0.339	[−0.37, 0.80]	−0.04	0.416	[−0.49, 0.46]
Extraversion									
Uncontrolled	−0.85	0.015	[−1.74, 0.05]	−0.34	0.297	[−1.20, 0.51]	−0.52	0.014	[−1.17, 0.11]
Controlled	−1.43	0.134	[−3.90, 1.03]	−0.65	0.356	[−2.47, 1.17]	−0.58	0.075	[−1.72, 0.54]
Agreeableness									
Uncontrolled	−0.11	0.509	[−0.52, 0.31]	0.04	0.815	[−0.40, 0.48]	−0.09	0.290	[−0.49, 0.35]
Controlled	−0.10	0.610	[−0.61, 0.41]	0.04	0.813	[−0.43, 0.51]	−0.11	0.256	[−0.55, 0.34]
Neuroticism									
Uncontrolled	−0.40	0.073	[−0.97, 0.17]	−0.02	0.926	[−0.51, 0.47]	−0.38	0.031	[−0.91, 0.15]
Controlled	−0.42	0.114	[−1.10, 0.26]	−0.09	0.685	[−0.67, 0.49]	−0.37	0.074	[−1.05, 0.34]

*Note:* Bold estimates are significant at *p* < 0.01. Uncontrolled = No covariates included. Controlled = Models include gender, HISEI, and fluid reasoning as individual, and school track as class level covariates. Doubly Latent = maximum likelihood estimation of doubly latent contextual models. Manifest Latent = maximum likelihood estimation of manifest‐latent contextual models. Bayesian = Bayesian estimation of doubly latent contextual models.

Regarding the covariates, student's sex robustly predicted individual change in extraversion (*b*s = −0.08, *p*s ≤ 0.001), agreeableness (*b*s = 0.04–0.13, *p*s ≤ 0.006), and neuroticism (*b*s = 0.26–0.44, *p*s ≤ 0.006). Girls reported lower levels of extraversion and higher levels of agreeableness and neuroticism at the second measurement point. Furthermore, the school track emerged as a robustly significant predictor of class level conscientiousness (*b*s = −0.20 to −0.14, *p*s ≤ 0.001), indicating that classes at academic track schools reported lower levels of conscientiousness on average compared to classes at nonacademic track schools.

Given the absence of the expected composition effects regarding conscientiousness and the school track differences in class levels of this trait, we decided to further explore the relevance of school track differences regarding the composition effects in our sample. To do so, we set up a series of multigroup models based on the preregistered doubly latent modeling approach to test whether the estimates for the composition effects for all Big Five traits varied across school tracks. Replicating our main findings, the composition effect for openness reached significance. Interestingly, this was only the case for academic track schools (*b* = −0.57, *p* = 0.001), but not for nonacademic track school (*b* = −0.07, *p* = 0.734). This result pattern remained robust after including the individual level covariates gender, parental socioeconomic status, and fluid reasoning. The expected composition effect regarding conscientiousness again remained insignificant (see Table S5 for detailed results).

Taken together, the results of Study 1 do not support our assumption of the relevance of peer composition effects for personality development in the school contexts. Our results show a relatively high rank‐order stability of students' personality from 7th to 9th grade. Against our expectation, the significant composition effect of openness in classrooms was negative, which is contrary to previous findings in university learning groups (Shan and Zölitz 2023). This opposite direction might indicate that social comparison, as compared to socialization, processes are more pronounced within the classroom context. To illustrate, being constantly surrounded by and learning in a classroom with classmates who report higher levels of intellectual curiosity, read a lot, and regularly visit museums (e.g., high openness), might trigger contrasting social comparison processes that, in the long run, lead to a more negative evaluation of a student's own intellectual curiosity. However, the composition effect regarding openness did not emerge robustly across modeling strategies and, thus, should be interpreted cautiously. At the same time, our exploratory analyses indicate, that composition effects regarding openness might vary across school tracks and are more pronounced in particularly intellectual challenging contexts such as academic track schools.

Despite having a large and diverse sample, at least one limitation characterizes our Study 1. Despite its frequent application particularly in panel studies, the very short personality measure in the NEPS study has the limitations of an incomplete representation of the broadness of the underlying traits and the relatively low reliability coefficients. While we controlled for measurement error by using a diverse set of latent variable approaches, the brevity of the measure limited its validity to a certain extent. Therefore, we aimed to replicate our results in Study 2 using an independent adolescent sample and applying a longer and more reliable personality measure.

## 7 Study 2: Method

Within Study 2, we used existing data from the SEED project. The SEED study tracked 10th grade students across one school year as well as their transition to 11th grade (upper secondary school). The study included four measurement time points with roughly quarterly intervals. The present paper uses data from the first (T1) and third (which will be called T2 from now on) measurement point only, which were assessed at the beginning/middle (November 2022 to February 2023) and the end (June 2023) of the 10th grade, respectively. More information on the SEED data collection is available at the OSF project page (https://osf.io/xyf8c/).

### 7.1 Sample

Study 2 was based on a total sample of *N* = 788 tenth graders nested in 54 classes from 12 schools meeting the inclusion criteria of at least one answered personality item at T1 and an available class identification. There were on average *M* = 14.28 (*SD* = 5.12, Range: 1^7^–26) participants in each class. The majority of participants attended vocational‐track schools (43.9%), which prepare for vocational training, while all other participants attend academic‐track schools (56.1%), which prepare students for university education. On average, students were 15.49 (*SD* = 0.68) years old at T1, with an equal distribution of girls and boys (49.6% female). Most participants reported that at least one parent had an intermediate (49.7%) or high (47.0%) educational qualification as categorized by CASMIN Educational Classification scheme (Brauns, Scherer, and Steinmann 2003). An immigrant background was reported by 47.3% of the participants.

### 7.2 Measures and Procedure

#### 7.2.1 Big Five Personality Traits

Within Study 2, Big Five personality was assessed using the well‐established and reliable German BFI‐2 (Danner et al. 2016) again at T1 and T2. The BFI‐2 assesses the Big Five traits with 12 items (4 items per facet) each. All items were rated on a 7‐point scale ranging from 1 (*does not apply at all*) to 7 (*fully applies*). We included personality measures from T1 and T2 in our analyses. The reliability of the measurement models was sufficient (*ω*_opennessT1/T2_ = 0.81/0.86, *ω*_*c*onscientiousness T1/T2_ = 0.87/0.87, *ω*_extraversion T1/T2_ = 0.87/0.87, *ω*_agreeableness T1/T2_ = 0.82/0.86, ω_neuroticism T1/T2_ = 0.87/0.88). To account for the unreliability of the observed items, we used latent variable models for all Big Five traits.

#### 7.2.2 Control Variables

Control variables are all considered from T1. S*chool track* (0 = nonacademic; 1 = academic) and *gender* (0 = male, 1 = female) served as manifest control variables at the class and individual level, respectively. Moreover, we included *fluid reasoning* assessed with 12 items arranged in 3 sets of 4 items retrieved from the international cognitive ability resource (Condon and Revelle 2014) as a control variable at the individual level. The sets comprised letter series, matrix, and verbal reasoning tasks. We used the sum score of these items as a manifest covariate. Additionally, the highest parental education which was assessed by two items (for mothers and fathers, separately) served as a manifest indicator of *socioeconomic status* at the class and individual level. Parental education was categorized according to the CASMIN Educational Classification index (Brauns, Scherer, and Steinmann 2003). That is, high education (CASMIN 3) refers to university degrees; intermediate education (CASMIN 2) includes secondary school certificates and comparable qualifications; and low education (CASMIN 1) subsumes no formal qualification as well as lower secondary education. These levels were recoded so that 0 referred to low, 1 to intermediate, and 2 to high education.

### 7.3 Statistical Analyses

We followed an identical modeling approach as described in Study 1 testing three modeling strategies (Lüdtke et al. 2008; Marsh et al. 2009): (1) doubly latent contextual models, (2) manifest‐latent contextual models, and 3) the Bayesian modeling approach to estimate doubly latent contextual models. There is one difference in model specification regarding the doubly latent models: Given the more extensive personality measure assessed in Sample 2, we specified the Big Five traits again as latent constructs but now follow a content‐based parceling approach (e.g.; Matsunaga 2008). Thus, we used the three facet means as the three indicators for each trait. Model outputs can be found at the OSF project page (https://osf.io/evga9/).

## 8 Study 2: Results and Discussion

Similar to Study 1, intraclass correlation coefficients (0.01–0.08) indicated a low amount of variance attributable to the class level (see Table 4). Thus, again only between 1% and 8% of the variance in Big Five personality traits could be attributed to differences between classrooms and the remaining amount of variance is due to interindividual differences between students within the same classroom. Table 5 presents manifest correlations between the Big Five personality traits at both measurement points and all covariates. Across all three modeling approaches, our models with and without covariates fit the data well (see Table S7). Again, we apply the same reporting strategy as described for Study 1.

**TABLE 4:** Study 2—intraclass correlation coefficients (ICCs) and cluster variances for personality scales.

	ICC	var_within_	var_between_
Openness T1	0.08	0.80	0.07
Conscientiousness T1	0.01	1.09	0.02
Extraversion T1	0.02	0.98	0.02
Agreeableness T1	0.05	0.73	0.04
Neuroticism T1	0.01	1.05	0.01

Abbreviations: T1, first measurement at the start of 10th grade; var_between_, variance between classes; var_within_, variance within classes.

**TABLE 5:** Study 2—full sample descriptives (means, *SD*s, number of observations, and correlations of big five traits, sociodemographic characteristics and covariates).

Variable	*M*	*SD*	*N*	1	2	3	4	5	6	7	8	9	10	11	12	13	14
1. Openness T1	4.64	0.93	788														
2. Openness T2	4.58	0.97	258	**0.74**													
3. Conscientiousness T1	4.42	1.00	788	**0.21**	0.15												
4. Conscientiousness T2	4.30	0.94	258	0.11	**0.20**	**0.81**											
5. Extraversion T1	4.48	1.03	788	**0.35**	**0.27**	**0.25**	0.13										
6. Extraversion T2	4.46	0.98	258	**0.28**	**0.30**	**0.23**	**0.21**	**0.79**									
7. Agreeableness T1	4.78	0.87	788	**0.18**	0.10	**0.35**	**0.27**	0.08	−0.01								
8. Agreeableness T2	4.74	0.91	258	0.11	**0.18**	**0.25**	**0.34**	0.04	0.06	**0.79**							
9. Neuroticism T1	4.17	1.05	788	−0.04	0.03	**−0.34**	**−0.26**	**−0.24**	−0.14	**−0.20**	−0.13						
10. Neuroticism T2	4.05	1.02	258	0.04	−0.01	**−0.22**	**−0.32**	**−0.20**	**−0.20**	−0.06	−0.14	**0.77**					
11. Female	0.50	0.50	788	**0.16**	**0.23**	**0.11**	0.07	0.04	0.11	**0.20**	**0.25**	**0.38**	**0.40**				
12. Fluid Reasoning T1	5.71	2.74	778	0.05	−0.02	**−0.10**	−0.04	0.00	−0.00	0.04	0.09	−0.01	−0.12	0.01			
13. School Track	0.56	0.50	788	0.07	−0.00	**−0.11**	−0.02	0.09	0.13	**0.11**	**0.17**	0.06	−0.08	0.08	**0.50**		
14. Parental Education	1.44	0.56	423	0.12	−0.08	−0.05	−0.01	0.06	−0.04	−0.04	0.01	0.08	0.06	0.01	**0.23**	**0.28**	
15. Immigrant Background	0.47	0.50	788	0.01	0.04	**0.11**	0.10	0.04	−0.03	−0.03	0.01	−0.06	−0.10	−0.07	**−0.16**	**−0.15**	−0.05

*Note:* Significant correlations (*p* < 0.01) are in bold. Sample sizes vary across correlations from 153 to 788. School track is dummy coded with 1 = academic track. Parental Education is highest educational degree of both parents differentiated according to the CASMIN scale (Brauns, Scherer, and Steinmann 2003) ranging from 0 to 2. Immigrant background is dummy coded with 1 = immigrant background.

Regarding the rank‐order stability of the Big Five traits at the individual level, results were similar across all modeling approaches revealing relatively high stability (*b*s: 0.74–0.89, *p*s < 0.001). Again, the stability estimates were robust for the addition of covariates (see Tables S8 and S9 for detailed results). Thus, results indicated that the Big Five traits were highly stable at the individual level across the school year in 10th grade.

Regarding the composition effects, estimates obtained from the different modeling approaches are presented in Table 6. Across all three modeling approaches and across models without and with covariates, no significant compositions effects emerged contradicting our hypotheses. The class level average of the Big Five traits did not predict differences in individual personality change across one school year. Regarding the covariates, girls again reported higher levels of neuroticism (*b*s = 0.22–0.27, *p*s < 0.001). Furthermore, classes at academic track schools reported lower levels of neuroticism (*b*s = −0.15 to −0.14, *p*s ≤ 0.008). Again, we ran exploratory multigroup models following the doubly latent modeling approach. No significant composition effects emerged across school tracks (see Table S10 for detailed parameter estimates).

**TABLE 6:** Study 2—composition effect estimates across modeling approaches.

	Doubly Latent			Manifest Latent			Bayesian		
	Estimate	*p*	99% CI	Estimate	*p*	99% CI	Estimate	*p*	99% CI
Openness									
Uncontrolled	−0.46	0.076	[−1.12, 0.21]	−0.24	0.270	[−0.80, 0.32]	−0.35	0.144	[−1.31, 0.59]
Controlled	−0.47	0.122	[−1.26, 0.31]	−0.26	0.264	[−0.84, 0.33]	−0.33	0.188	[−1.43, 0.74]
Conscientiousness									
Uncontrolled	−0.06	0.708	[−0.47, 0.35]	−0.07	0.757	[−0.65, 0.51]	−0.09	0.390	[−1.04, 0.82]
Controlled	0.13	0.557	[−0.46, 0.72]	0.15	0.707	[−0.89, 1.19]	−0.08	0.439	[−6.53, 2.17]
Extraversion									
Uncontrolled	−0.76	0.042	[−1.71, 0.20]	−0.62	0.133	[−1.68, 0.44]	−0.31	0.247	[−1.59, 1.00]
Controlled	−1.04	0.014	[−2.13, 0.05]	−0.93	0.175	[−2.69, 0.84]	−0.48	0.292	[−7.05, 5.40]
Agreeableness									
Uncontrolled	0.16	0.653	[−0.74, 1.06]	0.30	0.373	[−0.58, 1.19]	0.12	0.341	[−0.72, 1.08]
Controlled	0.17	0.633	[−0.74, 1.08]	0.33	0.373	[−0.62, 1.28]	0.07	0.441	[−2.44, 2.11]
Neuroticism									
Uncontrolled	−0.20	0.751	[−1.78, 1.39]	0.07	0.865	[−0.93, 1.06]	−0.21	0.320	[−1.58, 1.18]
Controlled	−0.14	0.837	[−1.87, 1.60]	0.22	0.569	[−0.79, 1.24]	−0.14	0.409	[−4.08, 2.14]

*Note:* Bold estimates are significant at *p* < 0.01. Uncontrolled = No covariates included. Controlled = Models include gender, HISEI, and fluid reasoning as individual, and school track as class level covariates. Doubly Latent = Maximum likelihood estimation of doubly latent contextual models. Manifest Latent = Maximum likelihood estimation of manifest‐latent contextual models. Bayesian = Bayesian estimation of doubly latent contextual models.

Taken together and similar to Study 1, the results of Study 2 do not support the relevance of personality peer composition effects for individual personality development across 10th grade. This is contrary to our hypothesis regarding the relevance of class‐level openness and conscientiousness and to initial results in university learning contexts supporting the relevance of peer personality (Shan and Zölitz 2023). Still, the results again point towards a relative stability of the Big Five traits across the 10th grade and little systematic personality differences between classrooms.

## 9 General Discussion

Based on two large German samples spanning two distinct time frames, we investigated the relevance of class‐level personality for individual personality development. The results revealed three main findings: First, from 7th to 9th grade and from the beginning to the end of 10th grade, individual‐level personality showed high stability. Second, class‐level openness and conscientiousness were not robustly linked to individual personality development, and the only found effects were opposite to our hypotheses. Specifically, students in classes with higher average levels of openness experienced a decrease in openness to experience but only in one of our samples. Third, our analyses of the remaining Big Five traits revealed no additional composition effects. In the following, we will draw theoretical implications from these findings and discuss the methodological challenges of investigating personality composition effects.

### 9.1 Classrooms as Reference Groups for Personality Development

Given that schools and classrooms are key developmental contexts in adolescence and are strongly characterized by peer interactions (Eccles and Roeser 2011), we investigated the relevance of class‐average peer personality for individual personality development. Previous educational research has established the relevance of peer characteristics within educational settings for learning outcomes and socioemotional characteristics (Belfi et al. 2012; Fang et al. 2018; Van Ewijk and Sleegers 2010). Theoretically, these peer composition effects may arise from different psychological processes including social comparison and socialization processes (Dreeben and Barr 1988; Thrupp, Lauder, and Robinson 2002). First findings based on adult samples indicated both the relevance of social comparison for personality self‐reports (Lenhausen, Bleidorn, and Hopwood 2023; Lenhausen, Hopwood, and Bleidorn 2023) and socialization processes for individual personality development within educational peer groups (Shan and Zölitz 2023) thereby implying that the rationale of composition effects in schools and classrooms might reasonably apply to individual personality development.

Given the similarity of the learning contexts, we wanted to test whether the socialization effects reported by Shan and Zölitz (2023) could be transferred to adolescents within the context of school classrooms. More specifically, we hypothesized that higher classroom levels of openness and conscientiousness should be associated with more pronounced individual gains in these traits across time. However, our results did not support this hypothesis. In contrast, there were no robust indications of classroom composition effects on individual Big Five development. Moreover, the composition effect of openness to experiences was even in the opposite direction in one sample. Thus, individual students did not benefit from higher classroom levels of openness but they contributed to a more negative developmental trend.

#### 9.1.1 Theoretical and Methodological Reasons for Unexpected Result Pattern

What are potential reasons for the unexpected result patterns? There are theoretical and methodological explanations for the absence of the hypothesized effects in our study. First, theoretically the absence of robust personality composition effects in our sample might indicate the irrelevance of peers in the classroom for individual personality development. While our results indeed do not rule out the absence of classroom peer effects, we believe the absence contradicts previous research giving both theoretical and empirical support for the relevance of peers for personality self‐reports (Lenhausen, Bleidorn, and Hopwood 2023; Lenhausen, Hopwood, and Bleidorn 2023) and personality development in adolescence (e.g., Israel et al. 2023). However, given the absence of clear composition effects in both of our classroom samples, it might be possible that the different psychological processes, expected to be involved in composition effects (Dreeben and Barr 1988; Thrupp, Lauder, and Robinson 2002), might have counteracted each other at the classroom level. That is, there is support for the relevance of social comparison for personality self‐perceptions (Credé, Bashshur, and Niehorster 2010; Lenhausen, Bleidorn, and Hopwood 2023; Lenhausen, Hopwood, and Bleidorn 2023) as well as a first study supporting peer socialization effects for trait development (Shan and Zölitz 2023). While peer comparison processes imply a negative correlation, peer socialization processes imply a positive association between the expression of a characteristic at the group versus the individual level. Therefore, the joint appearance of both processes might explain the absence of robust findings in our study.

Second, the unexpectedly negative composition effect regarding openness might indicate the importance of social comparison processes within classrooms at least to some degree. As openness is known to be positively related to cognitive ability (Anglim et al. 2022), composition effects on openness self‐perceptions might be more similar to those on ability self‐perceptions (e.g., academic self‐concepts) which are known to illustrate social comparison rather than socialization effects (Fang et al. 2018). That is, students seem to contrast their level of open‐minded behavior with that of their classmates and use their classroom as a feedback and reference group for their self‐reports (Credé, Bashshur, and Niehorster 2010; Lenhausen, Bleidorn, and Hopwood 2023; Lenhausen, Hopwood, and Bleidorn 2023). However, the negative composition effect regarding openness should be interpreted cautiously, as it did not emerge robustly across samples and modeling strategies.

Third, research on acad other self‐related constructs also indicates that the formation of self‐related beliefs involves not only social comparison processes but also self‐related comparisons. For example, the formation of academic self‐concepts involves comparing one's abilities to their classmates' abilities as well as their own previous abilities (e.g., Trautwein and Möller 2016; Wolff et al. 2018). While we did not specifically address self‐related comparisons within our study, it could be that given the increasing self‐reflection abilities in adolescence, self‐related comparisons are more relevant to the development of Big Five personality traits in this period.

Fourth, it is important to note that academic self‐concepts conceptually differ from the Big Five personality in terms of their specificity. In contrast to the Big Five traits reflecting broad behavioral tendencies across domains and contexts, academic self‐concepts refer to self‐perceptions of academic abilities that are specific to educational contexts and/or even academic domains. Furthermore, research designs investigating classroom effects on academic self‐concepts often involve more objective class‐level predictors (e.g., standardized ability tests). These measures are less affected by possible class‐specific frame‐of‐reference effects given that they are distinct from the respective self‐perception. Accordingly, future research on class composition effects on individual personality development could benefit from using more context‐specific or objective measures of classmates' typical behavioral tendencies.

Fifth, another potential reason for the absence of the expected results are the low ICCs of our personality data. That is, the average levels of Big Five personality traits differed only to a very limited amount across classrooms. Rather most variance in Big Five traits was attributable to individual differences within classes. While this was not unexpected and this kind of variance distribution is comparable to those of other psychological constructs within educational contexts (Martin et al. 2011), it poses a conceptual and methodological challenge. On the one hand, the small differences in average levels of personality traits between classes could actually reflect potential reference group effects, even referred to as “biases” in self‐reports within classes (Lira et al. 2022). That is, students' personality self‐reports potentially reflect their perceived level of the Big Five traits relative to their class, rather than relative to all students their age. However, we were not able to directly test such effects in our study due to the research design. On the other hand, the low ICCs impose a methodological challenge as they reduce the possibility of finding potential effects of class‐level personality within our correlational study design. That is, the power of the statistical models is partly a function of the ICC of the construct under investigation (for further information on power see also Section 9.2).

Finally, the high rank‐order stabilities of personality are a second methodological issue that further complicate the statistical detection of composition effects. Although the stability estimates were somewhat higher and more consistent across modeling strategies in Study 2 as compared to Study 1, all Big Five traits were highly stable from 7th to 9th grade (Study 1) and across 10th grade (Study 2). Study differences are most likely due to design differences, with Study 1 spanning a longer time interval and using a shorter personality measure. Longer time intervals typically result in lower stability estimates as they potentially allow for more changes to occur as more time passes. The short personality scale is less representative of the broadness of the traits. Thereby it is more sensitive to minor changes in response behavior or the specific behaviors captured by the two items per trait. The higher relative weight of each item could contribute to the lower stability of the traits, especially in manifest models that do not control for measurement error (Brandt et al. 2023). In addition, the difference in the robustness of the stability estimates potentially illustrates an age effect between middle and later adolescence. That is, previous research has shown that the Big Five traits become increasingly stable across adolescence (Borghuis et al. 2017). Therefore, the older average age of the students in Study 2 might have contributed to the higher stability of the Big Five traits.

#### 9.1.2 Characteristics of the Research Design and Composition Effects

What are potential differences in research design that contribute to differential composition effects? We would like to discuss four differences between the research designs used by Shan and Zölitz (2023) and in our study that are potentially relevant to the investigation of composition effects: group size, group function, time of group initiation, and the broader contextual setting. First, our study differed from the study of Shan and Zölitz (2023) regarding the group size. Whereas they based their study on small learning groups of only four students, our investigation focused on classes comprising up to 31 students. One might argue that not all classmates are equally important for an individual's personality development. It could be that the class average of certain traits does not sufficiently reflect the trait levels of the peers most relevant to one individual (e.g., friends, seatmates). Therefore, potential socialization processes at more specific peer levels might have been masked at the class level due to the large group size and should be specifically targeted in future research.

Second, the impact of the peer group on an individual's perception of specific traits might vary not only with group size but also with group function. The learning groups studied by Shan and Zölitz (2023) served a rather specific academic function potentially resulting in more pronounced composition effects indicating socialization processes regarding the academically relevant traits of openness and conscientiousness (Mammadov 2022). To illustrate, it seems reasonable that an individual adapts the typical academic behavior of their learning group peers as they regularly share their learning experiences and thus their typical learning behavior. By contrast, our study involved classroom peers who not only share academic experiences but experience various (social) interactions across the day with different classroom peers that do not exclusively serve an academic function. In line with such arguments, the average class level of openness and conscientiousness in a classroom might be less explanatory for an individual's development in these traits compared to the average group levels in small, focused learning groups at university. To illustrate, students might rather infer something about their academic behavior based on specific others that they share academic interactions with. Within the school context, such specific others might be close friends who students select as study partners or classmates with whom they work together at a given task rather than all classmates.

Third, the study of Shan and Zölitz (2023) assessed initial personality directly at the beginning of the group interaction, that is, their first measurement directly preceded the group initiation phase. On the contrary, in our two samples, the groups were existing classrooms that neither changed nor were randomly assigned at the beginning of the studies. This difference in study settings might explain the absence of most and the opposite direction of some of the hypothesized composition effects. On the one hand, positive socialization processes might be most pronounced in the specific period following group initiation. In this sensitive period at the beginning, individuals might be more susceptible to group information due to their desire to become part of the group (Deci and Ryan 2002). On the other hand, different peer processes might occur at differing time points after the initiation of a group. Within already existing peer groups such as classrooms, it could be possible that social comparison processes might be more prevalent than socialization. This could result in the negative association of class‐average openness with individual development of this trait in our sample. That is, students rather downgrade their intellectual curiosity due to the many more open behaviors of classmates. Unfortunately, our research design did not allow us to disentangle the different processes involved in peer composition effects. Thus, future research should adopt methodological approaches enabling a more differentiated picture of composition effects on personality development.

Fourth, our exploratory analyses indicated that the broader contextual setting, that is, the school track potentially plays a role in (classroom) composition effects. Specifically, in Study 1 the negative composition effect of openness was only present at academic track schools but not at nonacademic track schools. This finding suggests that characteristics of the general school context further differentiate classroom composition effects on personality development. Potential reasons for more pronounced effects at academic track schools might be differences in the composition, organization, and expectations across school tracks in the German school system (Brandt et al. 2020). Tracking practices in Germany are mostly based on ability. Given the associations between openness and cognitive abilities (Anglim et al. 2022), social comparison effects on openness might be specifically pronounced in academic environments with higher cognitive ability levels. As for organizational reasons, in academic track schools students mostly share their lessons with the same classmates, whereas in nonacademic track schools, they often practice course‐by‐course tracking (Becker, Neumann, and Dumont 2017). This course‐by‐course tracking softens the definition of classrooms, potentially making it harder to find clear composition effects. As for values and expectations, there is first evidence indicating that personality performance associations differ between school tracks (Brandt et al. 2020). These differences most likely reflect distinct representations of learning environments that result in different demands and opportunities for trait expression and, thus, in possibilities for perceiving the traits of classroom peers. Thus, it might be possible that different school tracks involve distinct peer processes affecting personality development.

### 9.2 Limitations and Future Research

While our study was based on two independent large‐scale samples and we applied three state‐of‐the‐art methodological approaches to investigate the role of class‐average personality for individual personality development, the following limitations should be considered when interpreting the results and developing future studies. First, while our power simulations suggest that our samples were appropriately sized to detect a range of effect sizes, it is important to note that the power to detect small class‐level effects, particularly in Study 2, was considerably lower. Given the low ICCs, the NEPS sample provided sufficient power to detect small to medium (*b* = 0.20) class‐level effects, while the SEED sample did so only for medium (*b* = 0.50) class‐level effects. This discrepancy in power might partly explain why the significant effect of class‐level openness, identified in Study 1, did not replicate in Study 2. However, we also need to point out that our power simulations were based on manifest modeling approaches. Existing literature suggests that latent variable modeling—which accounts for measurement error and captures underlying constructs more effectively—may offer higher statistical power than the manifest approaches we applied (Wolf et al. 2013). Therefore, the results of our simulations represent the lower bounds of the actual power achieved, and we consider our analyses to be sufficiently powered overall.

Second, the correlational design of our study does not allow for causal interpretations. Despite using a longitudinal design and including relevant covariates, we cannot rule out that the associations between initial class level and changes in individual traits were mediated or masked by other (unobserved) variables or driven by selection effects. For example, there might be additional variables shared by classes affecting the individual personality development such as teaching characteristics, performance feedback, or school policy differences. At the same time, studies in naturally existing groups are typically characterized by such correlational designs as any form of randomization, particularly in educational contexts, is often impractical.

Third, while we based our analyses on two samples with different measurement intervals, there might not have been enough variation in personality changes across these intervals. Future research should consider various time points and measurement intervals to gain a more comprehensive view of potential peer composition effects on personality development. To illustrate, it might be insightful to study classroom composition effects right after the transition to secondary school, given the new formation of classes at this time point. Such natural changes in class composition might be best suited to address the relevance of peer composition utilizing a quasi‐experimental design. Furthermore, it appears to be important to assess personality not just across this sensitive period, but also at several time points after such a transition, because the timing of classroom composition effects is not yet well understood.

Fourth, the measurement of personality has two important limitations: On the one hand, we used self‐report measures in both studies. As we were interested in the relevance of peer personality for the development of an individual's self‐perception, self‐reports are an informative outcome measure. However, additional personality measures such as other reports or behavioral data might help to deepen the understanding of class‐level personality and disentangle the processes involved in peer composition effects, as they potentially allow for a differentiation between changes in observable behavior and self‐related cognitions. On the other hand, the shortness of the personality scale in our Study 1 imposed certain challenges in estimating our models. That is, although this is a widely used and established Big Five scale, particularly its brevity might reduce the potential to find school‐specific associations with personality change (Johannsen et al., in press).

## 10 Conclusion

Taken together, our study provided the first empirical test of peer composition effects on individual personality development. Across two large adolescent samples, evidence for the effects of the average classroom personality on a student's personality development was weak or even absent. In the light of highly stable traits across relatively short time intervals, a singular significant effect of class‐level personality on openness was found that might hint at the relevance of composition effects for trait development. Future research should explore potential processes involved in peer composition effects on personality development by systematically varying peer group size, function, and different states of peer group formation.

## Author Contributions

M.J. played a lead role in conceptualization, data curation, formal analysis, methodology, project administration, writing of original draft, and editing. N.D.B. played a supporting role in supervision, methodology as well as writing and editing. O.L. played a supporting role in methodology, writing and editing. J.W. played a lead role in funding acquisition, resources, and supervision as well as a supporting role in conceptualization, methodology, project administration, writing of the original draft, and editing.

## Ethics Statement

The ongoing NEPS study (Study 1) is conducted under the supervision of the German Federal Commissioner for Data Protection and Freedom of Information (BfDI) and all procedures are approved by the data protection unit of the Leibniz Institute for Educational Trajectories (LIfBi). The SEED project (Study 2) was approved by the local school authority and the local ethics committee of the psychological institute of the University of Hamburg (2022_45).

## Conflicts of Interest

The authors declare no conflicts of interest.

## Supporting information


Data S1.

## Data Availability

The data that support the findings of Study 1 are available from the NEPS. Data are available at https://www.neps‐data.de/Data‐Center/Data‐Access with the permission of the Leibniz Institute for Educational Trajectories. The data that support the findings of Study 2 are openly available in the OSF at https://osf.io/evga9/.

## Endnotes

^1^
Given the complexity of assumptions necessary for power simulation for SEM approaches, we used power simulations for manifest models as a lower boundary for the power estimate for our study (Wolf et al. 2013).

^2^
We already conducted preliminary power simulations as part of the preregistration (see OSF project page) but updated the simulations based on actual sample sizes during the writing of this manuscript.

^3^
As the class codes available within the NEPS data are not linked across waves, longitudinal class codes were obtained based on the procedure described by Florean, Hofmann, and Kleinert (2019). Moreover, the exclusion of participants changing classes was not preregistered but necessary based on our interest in class‐level personality.

^4^
As preregistered, we performed a robustness check excluding all classes with less than five students at T1. Result patterns were identical (cf., Table S6).

^5^
We preregistered only the first of the three described modeling approaches. Given we faced some estimation difficulties, we decided to strengthen the confidence in our results by testing the robustness of findings across different modeling approaches. We report all models and estimation procedures in the paper and on our osf page.

^6^
In deviation from our preregistration, we were not able to sufficiently estimate the models using the effect‐coding approach. Instead, we kept the models more parsimonious by restricting all loadings of the latent factors to 1 (see Table S1).

^7^
As preregistered, we performed a robustness check excluding all classes with less than five students at T1. Result patterns were identical (cf., Table S11).
