# Supplementary material for: Becoming as Open‐Minded and Organized as My Classmates? Peer Effects on Self‐Reported Personality Trait Development in the Classroom
Source: J Pers. 2025 Jan 10;93(6):1298–314. doi: 10.1111/jopy.13009 (PMC12592586; doi:10.1111/jopy.13009)
Supplement: Supplementary file 1 — Data S1. [file JOPY-93-1298-s001.pdf]

Becoming as Organized and Achieving as my Peers? Peer Effects on Self-Reported Personality Trait Development in the Classroom

Online Supplemental Materials

Table of Contents

Supplement A: Overall information

Table S1: *Data Driven Deviations from Preregistered Analysis Steps*

Supplement B: Study 1 – Additional Results

Table S2: *Study 1 - Model Fit of Multilevel Models Across Modelling Approaches*

Table S3: *Study 1 – Parameter Estimates of Uncontrolled Latent Multilevel Models*

Table S4: *Study 1 – Parameter Estimates of Controlled Latent Multilevel Models*

Table S5: *Study 1 - School Track Specific Composition Effects Obtained from Multigroup Doubly-Latent Contextual Multilevel Models*

Table S6: *Study 1 – Robustness Check (Exclusion of Classes < 5) of Composition Effect Estimates Obtained Across Modelling Approaches*

Supplement C: Study 2 – Additional Results

Table S7: *Study 2 - Model Fit of Multilevel Models Across Modelling Approaches*

Table S8: *Study 2 – Parameter Estimates of Uncontrolled Latent Multilevel Models*

Table S9: *Study 2– Parameter Estimates of Controlled Latent Multilevel Models*

Table S10: *Study 2 - School Track Specific Composition Effects Obtained from Multigroup Doubly-Latent Contextual Multilevel Models*

Table S11: *Study 2 – Robustness Check (Exclusion of Classes < 5) of Composition Effect Estimates Obtained Across Modelling Approaches*

Supplement A: Overall Information

Table S1

Data Driven Deviations from Preregistered Analysis Steps

Preregistered Analysis Step	Actual Analysis Step	Reason
**Overall changes**		
We preregistered to do our main analyses based on doubly-latent contextual models as proposed by Lüdtke and colleagues (Lüdtke et al., 2008; Marsh et al., 2009) using the robust maximum likelihood estimator (MLR; Yuan & Bentler, 2000).	We used not only the preregistered, but also two additional modelling approaches: manifest-latent contextual models (Lüdtke et al., 2008; Marsh et al., 2009) using the MLR estimator and doubly-latent contextual models using a Bayesian modelling approach (Zitzmann et al., 2016)	We were faced with some estimation difficulties (e.g. negative residual variances) and therefore decided to strengthen the confidence in our results by testing the robustness of findings across different modelling approaches.
We pre-registered to include our covariates as additional predictors in our main analyses.	We additional ran exploratory multi-group analyses, to further explore the impact of the school track on the composition effects.	Due to the absence of the expected results, we decide to take a deeper look into the relevance of the broader school environment by using a multigroup approach.
**Study 1**		
We preregistered to identify the measurement models using the effect coding method.	Instead, we kept the models more parsimonious by restricting all loadings of the latent factors to be equal to 1	We were not able to sufficiently estimate the models using the effect-coding approach and were faced with some modelling issues (e.g. negative residual variances and warning messages in some models). The adapted procedure enabled us to estimate all models without warning messages

Supplement B: Study 1 – Additional Results

Table S2

Model Fit of Multilevel Models Across Modelling Approaches

	Doubly-Latent					Manifest-Latent					Bayesian			
	N	CFI	RMSEA	AIC	BIC	*N*	CFI	RMSEA	AIC	BIC	*N*	CI	PPP	DIC
**Openness**														
Uncontrolled	5,461.00	1.00	0.01	52,891.08	52,996.76	5,440.00	1.00	0.00	26,236.57	26,289.38	5,461.00	[-19.36, 23.39]	0.43	52,929.55
Controlled	5,467.00	0.89	0.07	81,178.04	81,409.27	5,467.00	1.00	0.00	55,905.59	56,083.96	5,467.00	[227.40, 297.20]	0.00	81,197.02
**Conscientiousness**														
Uncontrolled	5,463.00	0.99	0.02	52,606.91	52,712.60	5,447.00	1.00	0.00	26,264.59	26,317.41	5,463.00	[-19.67, 23.90]	0.44	52,577.62
Controlled	5,467.00	0.98	0.03	81,003.92	81,235.15	5,467.00	1.00	0.00	56,088.23	56,266.61	5,467.00	[3.14, 64.18]	0.02	80,929.46
**Extraversion**														
Uncontrolled	5,460.00	0.99	0.02	52,433.31	52,538.99	5,417.00	1.00	0.00	26,165.78	26,218.56	5,460.00	[-17.91, 25.92]	0.36	52,417.19
Controlled	5,467.00	0.96	0.04	81,004.25	81,235.47	5,467.00	1.00	0.00	56,151.19	56,329.56	5,467.00	[21.67, 86.96]	0.00	80,900.16
**Agreeableness**														
Uncontrolled	5,463.00	0.98	0.01	80,921.97	81,073.90	5,417.00	1.00	0.00	26,595.12	26,647.90	5,463.00	[-24.09, 35.26]	0.34	80,894.93
Controlled	5,467.00	0.94	0.03	131,308.23	131,585.70	5,467.00	1.00	0.00	56,451.75	56,630.13	5,467.00	[18.62, 102.78]	0.00	109,242.65
**Neuroticism**														
Uncontrolled	5,466.00	0.99	0.02	53,958.63	54,064.33	5,435.00	1.00	0.00	26,836.61	26,889.42	5,466.00	[-19.92, 23.54]	0.45	54,005.55
Controlled	5,467.00	0.99	0.02	82,157.01	82,388.24	5,467.00	1.00	0.00	56,465.26	56,643.64	5,467.00	[-20.74, 39.09]	0.28	82,154.02

Note. Uncontrolled = No covariates included. Controlled = Models include sex, HISEI, and fluid reasoning as individual, and school track as class level covariates.

CFI = Comparative Fit Index, RMSEA = Root Mean Square Error of Approximation, AIC = Akaike Information Criterion, BIC = Baysian Information Criterion. CI = 95% confidence interval for the difference in the f statistic for real and replicated data, PPP = posterior predictive *p*-value, DIC = Deviance Information Criterion.

Table S3

Study 1 – Parameter Estimates of Uncontrolled Latent Multilevel Models

	DoublyLatent			ManifestLatent			Bayesian		
	b	*p*	99%-CI	*b*	*p*	99%-CI	*b*	*p*	99%-CI
**Openness**									
Openness$_{T2}$ on Openness$_{T1}$									
Within	**0.93**	< .001	[0.76, 1.09]	**0.51**	< .001	[0.47, 0.55]	**0.93**	< .001	[0.79, 1.09]
Between	**0.60**	< .001	[0.34, 0.87]	**0.58**	< .001	[0.31, 0.85]	**0.63**	< .001	[0.29, 0.98]
R^2 Openness$_{T2}$									
Within	**0.57**	< .001		**0.26**	< .001		**0.57**	< .001	
Between	0.88	.044		0.85	.054		**0.44**	< .001	
**Conscientiousness**									
Conscientiousness$_{T2}$ on Conscientiousness$_{T1}$									
Within	**0.72**	< .001	[0.63, 0.81]	**0.51**	< .001	[0.47, 0.55]	**0.72**	< .001	[0.64, 0.80]
Between	0.49	.014	[-0.03, 1.00]	0.53	.013	[-0.02, 1.08]	**0.60**	.002	[0.10, 1.08]
R^2 Conscientiousness$_{T2}$									
Within	**0.56**	< .001		**0.26**	< .001		**0.56**	< .001	
Between	0.29	.150		0.30	.137		**0.32**	< .001	
**Extraversion**									
Extraversion$_{T2}$ on Extraversion$_{T1}$									
Within	**1.04**	< .001	[0.88, 1.20]	**0.49**	< .001	[0.46, 0.53]	**1.03**	< .001	[0.90, 1.18]
Between	0.19	.568	[-0.67, 1.06]	0.15	.649	[-0.69, 0.99]	0.51	.016	[-0.12, 1.10]
R^2 Extraversion$_{T2}$									
Within	**0.60**	< .001		**0.24**	< .001		**0.60**	< .001	
Between	0.06	.760		0.04	.810		**0.20**	< .001	
**Agreeableness**									
Agreeableness$_{T2}$ on Agreeableness$_{T1}$									
Within	**0.60**	< .001	[0.48, 0.71]	**0.38**	< .001	[0.34, 0.42]	**0.60**	< .001	[0.50, 0.71]
Between	**0.49**	.001	[0.11, 0.87]	0.42	.012	[-0.01, 0.85]	**0.52**	< .001	[0.13, 0.93]
R^2 Agreeableness$_{T2}$									
Within	**0.39**	< .001		**0.14**	< .001		**0.40**	< .001	
Between	0.32	.059		0.22	.164		**0.26**	< .001	
**Neuroticism**									
Neuroticism$_{T2}$ on Neuroticism$_{T1}$									
Within	**0.79**	< .001	[0.60, 0.98]	**0.38**	< .001	[0.33, 0.42]	**0.80**	< .001	[0.64, 1.01]
Between	0.39	.053	[-0.13, 0.91]	0.36	.058	[-0.13, 0.84]	0.43	.013	[-0.06, 0.92]
R^2 Neuroticism$_{T2}$									
Within	**0.42**	< .001		**0.14**	< .001		**0.44**	< .001	
Between	0.49	.615		0.47	.666		**0.18**	< .001	

Table S4

Study 1 – Parameter Estimates of Controlled Latent Multilevel Models

	DoublyLatent			ManifestLatent			Bayesian		
	b	*p*	99%-CI	*b*	*p*	99%-CI	*b*	*p*	99%-CI
**Openness**									
Openness_{T2} on Openness_{T1}									
Within	**0.93**	< .001	[0.74, 1.11]	**0.48**	< .001	[0.44, 0.53]	**0.94**	< .001	[0.77, 1.14]
Between	**0.50**	< .001	[0.15, 0.86]	**0.44**	.002	[0.07, 0.81]	**0.55**	.002	[0.08, 1.02]
R^2 Openness_{T2}									
Within	**0.57**	< .001		**0.27**	< .001		**0.58**	< .001	
Between	0.79	.290		0.69	.406		**0.33**	< .001	
**Conscientiousness**									
$\text{Conscientiousness}_{T2}$ on $\text{Conscientiousness}_{T1}$									
Within	**0.70**	< .001	[0.61, 0.80]	**0.49**	< .001	[0.46, 0.53]	**0.70**	< .001	[0.63, 0.79]
Between	**0.68**	.001	[0.14, 1.22]	**0.71**	.001	[0.14, 1.28]	**0.67**	< .001	[0.22, 1.16]
R^2 $\text{Conscientiousness}_{T2}$									
Within	**0.57**	< .001		**0.26**	< .001		**0.57**	< .001	
Between	**0.68**	.001		**0.71**	< .001		**0.47**	< .001	
**Extraversion**									
Extraversion_{T2} on Extraversion_{T1}									
Within	**1.04**	< .001	[0.88, 1.20]	**0.49**	< .001	[0.46, 0.53]	**1.03**	< .001	[0.90, 1.19]
Between	-0.39	.681	[-2.85, 2.07]	-0.16	.820	[-1.98, 1.66]	0.45	.108	[-0.68, 1.58]
R^2 Extraversion_{T2}									
Within	**0.61**	< .001		**0.24**	< .001		**0.60**	< .001	
Between	0.53	.409		0.35	.299		**0.33**	< .001	
**Agreeableness**									
$\text{Agreeableness}_{T2}$ on $\text{Agreeableness}_{T1}$									
Within	**0.58**	< .001	[0.46, 0.70]	**0.37**	< .001	[0.33, 0.41]	**0.59**	< .001	[0.48, 0.70]
Between	0.48	.011	[-0.01, 0.96]	0.41	.022	[-0.05, 0.87]	**0.48**	.001	[0.06, 0.90]
R^2 $\text{Agreeableness}_{T2}$									
Within	**0.40**	< .001		**0.15**	< .001		**0.41**	< .001	
Between	0.28	.117		0.25	.108		**0.22**	< .001	
**Neuroticism**									
Neuroticism_{T2} on Neuroticism_{T1}									
Within	**0.69**	< .001	[0.50, 0.89]	**0.34**	< .001	[0.29, 0.38]	**0.71**	< .001	[0.54, 0.90]
Between	0.28	.263	[-0.36, 0.91]	0.24	.269	[-0.32, 0.81]	0.34	.066	[-0.32, 1.04]
R^2 Neuroticism_{T2}									
Within	**0.48**	< .001		**0.19**	< .001		**0.50**	< .001	
Between	0.30	.754		0.32	.771		**0.12**	< .001	

Table S5

Study 1 - School Track Specific Composition Effects Obtained from Multigroup Doubly-Latent Contextual Multilevel Models

	Uncontrolled			Controlled		
	b	*p*	95% CI	*b*	*p*	95% CI
			**Openness**			
non-academic	-0.07	.734	[-0.64, 0.50]	-0.13	.604	[-0.80, 0.53]
academic	**-0.57**	.001	[-1.00, -0.14]	**-0.60**	.001	[-1.06, -0.15]
			**Conscientiousness**			
non-academic	0.17	.671	[-0.86, 1.20]	0.19	.644	[-0.87, 1.25]
academic	-0.31	.250	[-0.99, 0.38]	-0.19	.437	[-0.81, 0.44]
			**Extraversion**			
non-academic	-0.67	.028	[-1.46, 0.12]	-0.67	.041	[-1.52, 0.17]
academic	-2.09	.020	[-4.40, 0.22]	**-2.46**	.009	[-4.90, -0.03]
			**Agreeableness**			
non-academic	-0.09	.603	[-0.53, 0.35]	-0.12	.497	[-0.59, 0.34]
academic	-0.04	.925	[-1.22, 1.13]	0.02	.975	[-1.52, 1.56]
			**Neuroticism**			
non-academic	-0.80	.073	[-1.94, 0.35]	-0.65	.166	[-1.87, 0.56]
academic	-0.11	.643	[-0.75, 0.52]	-0.23	.473	[-1.04, 0.58]

Note. Bold estimates are significant at $p < .01$. Non-academic = non-academic track schools, academic = academic track schools. Uncontrolled = No covariates included. Controlled = Models include sex, HISEI, and fluid reasoning as individual, and school track as class level covariates.

Table S6

Study 1 – Robustness Check (Exclusion of Classes < 5) of Composition Effect Estimates Obtained Across Modelling Approaches

	Doubly Latent			Manifest Latent			Bayesian		
	b	*p*	99% CI	*b*	*p*	99% CI	*b*	*p*	99% CI
	**Openness**								
Uncontrolled	**-0.34**	.007	[-0.67, -0.02]	0.05	.633	[-0.23, 0.33]	-0.31	.020	[-0.70, 0.08]
Controlled	**-0.44**	.005	[-0.84, -0.03]	-0.02	.866	[-0.37, 0.33]	-0.39	.025	[-0.89, 0.14]
	**Conscientiousness**								
Uncontrolled	-0.22	.307	[-0.78, 0.34]	0.04	.874	[-0.54, 0.62]	-0.11	.285	[-0.61, 0.44]
Controlled	-0.03	.902	[-0.60, 0.55]	0.22	.335	[-0.36, 0.79]	-0.02	.455	[-0.50, 0.48]
	**Extraversion**								
Uncontrolled	-0.82	.021	[-1.73, 0.09]	-0.34	.315	[-1.21, 0.53]	-0.51	.018	[-1.15, 0.13]
Controlled	-1.35	.141	[-3.70, 1.01]	-0.71	.379	[-2.81, 1.38]	-0.56	.070	[-2.31, 0.70]
	**Agreeableness**								
Uncontrolled	-0.17	.319	[-0.60, 0.26]	0.01	.974	[-0.45, 0.46]	-0.14	.195	[-0.59, 0.29]
Controlled	-0.15	.459	[-0.68, 0.38]	0.02	.923	[-0.47, 0.50]	-0.15	.180	[-0.62, 0.31]
	**Neuroticism**								
Uncontrolled	-0.41	.072	[-1.01, 0.18]	-0.03	.888	[-0.54, 0.48]	-0.40	.032	[-0.94, 0.17]
Controlled	-0.43	.106	[-1.13, 0.26]	-0.10	.675	[-0.69, 0.50]	-0.37	.076	[-1.06, 0.31]

Note. Bold estimates are significant at $p < .01$. Uncontrolled = No covariates included. Controlled = Models include sex, HISEI, and fluid reasoning as individual, and school track as class level covariates.
Doubly Latent = Maximum likelihood estimation of doubly-latent contextual models.
Manifest Latent = Maximum likelihood estimation of manifest-latent contextual models.
Bayesian = Bayesian estimation of doubly-latent contextual models.

Supplement C: Study 2 – Additional Results

Table S7

Study 2 – Model Fit of Multilevel Models Across Modelling Approaches

	**Doubly-Latent**					**Manifest-Latent**					**Bayesian**			
	N	*CFI*	*RMSEA*	*AIC*	*BIC*	*N*	*CFI*	*RMSEA*	*AIC*	*BIC*	*N*	*CI*	*PPP*	*DIC*
**Openness**														
Uncontrolled	788.00	0.96	0.07	8,142.49	8,249.88	788.00	1.00	0.00	2,756.97	2,794.32	788.00	[-20.56, 41.81]	0.28	8,137.21
Controlled	788.00	0.82	0.09	12,277.37	12,473.49	788.00	1.00	0.00	6,882.30	7,008.37	788.00	[62.28, 141.41]	0.00	12,265.01
**Conscientiousness**														
Uncontrolled	788.00	0.98	0.04	7,766.19	7,873.59	788.00	1.00	0.00	2,707.97	2,745.33	788.00	[-19.87, 43.74]	0.25	7,788.18
Controlled	788.00	0.94	0.05	11,898.71	12,094.83	788.00	1.00	0.00	6,834.93	6,961.01	788.00	[16.53, 97.10]	0.00	11,893.17
**Extraversion**														
Uncontrolled	788.00	0.99	0.04	7,649.63	7,757.02	788.00	1.00	0.00	2,736.01	2,773.37	788.00	[-22.05, 40.09]	0.32	7,679.64
Controlled	788.00	0.98	0.03	11,790.34	11,986.46	788.00	1.00	0.00	6,876.26	7,002.34	788.00	[-24.99, 54.45]	0.24	11,783.42
**Agreeableness**														
Uncontrolled	788.00	0.91	0.08	7,958.19	8,065.59	788.00	1.00	0.00	2,724.94	2,762.30	788.00	[-19.13, 44.69]	0.23	7,986.29
Controlled	788.00	0.87	0.07	12,056.94	12,253.06	788.00	1.00	0.00	6,832.19	6,958.27	788.00	[31.95, 113.49]	0.00	12,059.79
**Neuroticism**														
Uncontrolled	788.00	1.00	0.00	7,742.78	7,850.18	788.00	1.00	0.00	2,749.00	2,786.36	788.00	[-23.02, 39.22]	0.34	7,772.39
Controlled	788.00	1.00	0.00	11,728.56	11,924.67	788.00	1.00	0.00	6,759.58	6,885.66	788.00	[-30.71, 47.70]	0.34	11,739.39

Note. Uncontrolled = No covariates included. Controlled = Models include sex, HISEI, and fluid reasoning as individual, and school track as class level covariates.

CFI = Comparative Fit Index, RMSEA = Root Mean Square Error of Approximation, AIC = Akaike Information Criterion, BIC = Baysian Information Criterion. CI = 95% confidence interval for the difference in the f statistic for real and replicated data, PPP = posterior predictive *p*-value, DIC = Deviance Information Criterion.

Table S8

Study 2 – Parameter Estimates of Uncontrolled Latent Multilevel Models

	DoublyLatent			ManifestLatent			Bayesian		
	b	*p*	99%-CI	*b*	*p*	99%-CI	*b*	*p*	99%-CI
**Openness**									
Openness_{T2} on Openness_{T1}									
Within	**0.89**	< .001	[0.64, 1.13]	**0.74**	< .001	[0.59, 0.88]	**0.85**	< .001	[0.66, 1.06]
Between	0.43	.051	[-0.14, 0.99]	**0.50**	.007	[0.02, 0.98]	0.50	.064	[-0.44, 1.42]
R^2 Openness_{T2}									
Within	**0.68**	< .001		**0.54**	< .001		**0.67**	< .001	
Between	0.89	.672		0.87	.607		**0.23**	< .001	
**Conscientiousness**									
$\text{Conscientiousness}_{T2}$ on $\text{Conscientiousness}_{T1}$									
Within	**0.86**	< .001	[0.78, 0.94]	**0.81**	< .001	[0.74, 0.89]	**0.85**	< .001	[0.72, 0.98]
Between	**0.80**	< .001	[0.38, 1.21]	**0.74**	.001	[0.14, 1.35]	0.76	.014	[-0.17, 1.66]
R^2 $\text{Conscientiousness}_{T2}$									
Within	**0.80**	< .001		**0.66**	< .001		**0.80**	< .001	
Between	0.97	.091		0.85	.542		**0.41**	< .001	
**Extraversion**									
Extraversion_{T2} on Extraversion_{T1}									
Within	**0.85**	< .001	[0.75, 0.95]	**0.78**	< .001	[0.70, 0.86]	**0.86**	< .001	[0.73, 0.98]
Between	0.10	.798	[-0.86, 1.05]	0.16	.693	[-0.90, 1.22]	0.55	.114	[-0.73, 1.85]
R^2 Extraversion_{T2}									
Within	**0.74**	< .001		**0.62**	< .001		**0.75**	< .001	
Between	0.05	.959		0.05	.852		**0.21**	< .001	
**Agreeableness**									
$\text{Agreeableness}_{T2}$ on $\text{Agreeableness}_{T1}$									
Within	**0.91**	< .001	[0.78, 1.04]	**0.77**	< .001	[0.70, 0.84]	**0.90**	< .001	[0.72, 1.07]
Between	**1.06**	.002	[0.19, 1.94]	**1.07**	.002	[0.17, 1.97]	**1.02**	.001	[0.20, 1.95]
R^2 $\text{Agreeableness}_{T2}$									
Within	**0.76**	< .001		**0.61**	< .001		**0.74**	< .001	
Between	0.99	.676		0.98	.656		**0.60**	< .001	
**Neuroticism**									
Neuroticism_{T2} on Neuroticism_{T1}									
Within	**0.90**	< .001	[0.71, 1.09]	**0.81**	< .001	[0.67, 0.95]	**0.90**	< .001	[0.75, 1.05]
Between	0.70	.251	[-0.88, 2.29]	0.87	.026	[-0.14, 1.88]	0.69	.079	[-0.67, 2.06]
R^2 Neuroticism_{T2}									
Within	**0.74**	< .001		**0.62**	< .001		**0.74**	< .001	
Between	0.53	.902		0.67	.826		**0.29**	< .001	

Table S9

Study 2 – Parameter Estimates of Controlled Latent Multilevel Models

	DoublyLatent			ManifestLatent			Bayesian		
	b	*p*	99%-CI	*b*	*p*	99%-CI	*b*	*p*	99%-CI
**Openness**									
$Openness_{T2}$ on $Openness_{T1}$									
Within	**0.88**	< .001	[0.62, 1.15]	**0.73**	< .001	[0.56, 0.89]	**0.84**	< .001	[0.64, 1.08]
Between	0.42	.097	[-0.23, 1.06]	**0.48**	.010	[0.00, 0.96]	0.53	.074	[-0.58, 1.62]
R^2 $Openness_{T2}$									
Within	**0.69**	< .001		**0.54**	< .001		**0.68**	< .001	
Between	0.87	.694		0.83	.605		**0.31**	< .001	
**Conscientiousness**									
$Conscientiousness_{T2}$ on $Conscientiousness_{T1}$									
Within	**0.87**	< .001	[0.78, 0.96]	**0.82**	< .001	[0.74, 0.90]	**0.86**	< .001	[0.72, 1.01]
Between	**1.00**	< .001	[0.41, 1.59]	0.97	.015	[-0.05, 2.00]	0.80	.101	[-3.53, 5.19]
R^2 $Conscientiousness_{T2}$									
Within	**0.81**	< .001		**0.66**	< .001		**0.80**	< .001	
Between	0.98	.290		0.88	.746		**0.43**	< .001	
**Extraversion**									
$Extraversion_{T2}$ on $Extraversion_{T1}$									
Within	**0.84**	< .001	[0.75, 0.93]	**0.77**	< .001	[0.70, 0.84]	**0.85**	< .001	[0.72, 0.98]
Between	-0.20	.621	[-1.26, 0.85]	-0.16	.820	[-1.93, 1.61]	0.63	.227	[-3.85, 6.87]
R^2 $Extraversion_{T2}$									
Within	**0.74**	< .001		**0.62**	< .001		**0.76**	< .001	
Between	0.95	.214		0.84	.168		**0.56**	< .001	
**Agreeableness**									
$Agreeableness_{T2}$ on $Agreeableness_{T1}$									
Within	**0.89**	< .001	[0.74, 1.03]	**0.75**	< .001	[0.69, 0.81]	**0.88**	< .001	[0.71, 1.06]
Between	**1.06**	.001	[0.22, 1.90]	**1.08**	.004	[0.13, 2.03]	0.98	.082	[-3.22, 3.50]
R^2 $Agreeableness_{T2}$									
Within	**0.77**	< .001		**0.61**	< .001		**0.76**	< .001	
Between	0.99	.191		0.98	.195		**0.63**	< .001	
**Neuroticism**									
$Neuroticism_{T2}$ on $Neuroticism_{T1}$									
Within	**0.85**	< .001	[0.65, 1.05]	**0.76**	< .001	[0.62, 0.90]	**0.84**	< .001	[0.66, 1.01]
Between	0.70	.275	[-0.96, 2.37]	0.98	.012	[-0.02, 1.98]	0.70	.117	[-1.22, 2.86]
R^2 $Neuroticism_{T2}$									
Within	**0.76**	< .001		**0.63**	< .001		**0.75**	< .001	
Between	0.81	.870		0.85	.717		**0.33**	< .001	

Table S10

Study 2 - School Track Specific Composition Effects Obtained from Multigroup Doubly-Latent Contextual Multilevel Models

Track	Uncontrolled			Controlled		
	b	*p*	95% CI	*b*	*p*	95% CI
			**Openness**			
non-academic	-0.91	.064	[-2.18, 0.36]	-0.90	.231	[-2.83, 1.03]
academic	-0.29	.321	[-1.04, 0.46]	-0.29	.353	[-1.10, 0.52]
			**Conscientiousness**			
non-academic	-0.27	.054	[-0.64, 0.09]	-0.26	.063	[-0.63, 0.10]
academic	0.34	.040*	[-0.09, 0.77]	0.29	.055	[-0.10, 0.69]
			**Extraversion**			
non-academic	-0.92	.766	[-8.88, 7.05]	-1.45	.731	[-12.33, 9.43]
academic	-0.64	.382	[-2.54, 1.25]	-0.71	.431	[-3.05, 1.62]
			**Agreeableness**			
non-academic	-0.09	.546	[-0.45, 0.28]	-0.17	.248	[-0.55, 0.21]
academic	0.52	.380	[-1.01, 2.06]	0.54	.362	[-0.99, 2.08]
			**Neuroticism**			
non-academic	-0.59	.264	[-1.96, 0.77]	-0.60	.515	[-2.96, 1.76]
academic	0.18	.641	[-0.82, 1.18]	0.32	.891	[-5.74, 6.38]

Note. Non-academic = non-academic track schools, academic = academic track schools. Uncontrolled = No covariates included. Controlled = Models include sex, HISEI, and fluid reasoning as individual, and school track as class level covariates.

Table S11

Study 2 – Robustness Check (Exclusion of Classes < 5) of Composition Effect Estimates Obtained Across Modelling Approaches

	Doubly Latent			Manifest Latent			Bayesian		
	b	*p*	99% CI	*b*	*p*	99% CI	*b*	*p*	99% CI
					**Openness**				
Uncontrolled	-0.46	.070	[-1.12, 0.20]	-0.24	.266	[-0.80, 0.32]	-0.35	.146	[-1.31, 0.60]
Controlled	-0.48	.107	[-1.26, 0.29]	-0.26	.249	[-0.85, 0.32]	-0.33	.180	[-1.58, 0.70]
					**Conscientiousness**				
Uncontrolled	-0.07	.666	[-0.49, 0.35]	-0.08	.714	[-0.68, 0.51]	-0.10	.374	[-1.07, 0.86]
Controlled	0.13	.570	[-0.47, 0.74]	0.15	.722	[-0.91, 1.20]	-0.03	.474	[-2.64, 2.10]
					**Extraversion**				
Uncontrolled	-0.74	.050	[-1.70, 0.23]	-0.60	.151	[-1.69, 0.48]	-0.30	.244	[-1.66, 1.01]
Controlled	-1.01	.017	[-2.10, 0.08]	-0.92	.188	[-2.71, 0.88]	-0.31	.372	[-5.23, 16.59]
					**Agreeableness**				
Uncontrolled	0.16	.622	[-0.66, 0.97]	0.31	.332	[-0.51, 1.13]	0.12	.347	[-0.72, 1.09]
Controlled	0.17	.623	[-0.74, 1.08]	0.34	.370	[-0.63, 1.30]	0.11	.409	[-2.39, 3.15]
					**Neuroticism**				
Uncontrolled	-0.19	.756	[-1.78, 1.40]	0.07	.859	[-0.94, 1.07]	-0.23	.310	[-1.64, 1.16]
Controlled	-0.13	.855	[-1.89, 1.64]	0.23	.562	[-0.79, 1.25]	-0.10	.435	[-4.72, 6.44]

Note. Bold estimates are significant at $p < .01$. Uncontrolled = No covariates included. Controlled = Models include sex, HISEI, and fluid reasoning as individual, and school track as class level covariates.
Doubly Latent = Maximum likelihood estimation of doubly-latent contextual models.
Manifest Latent = Maximum likelihood estimation of manifest-latent contextual models.
Bayesian = Bayesian estimation of doubly-latent contextual models.
